# Under a New Light: Regulation of Light-Dependent Pathways by Non-coding RNAs

**DOI:** 10.3389/fpls.2018.00962

**Published:** 2018-07-26

**Authors:** Camila Sánchez-Retuerta, Paula Suaréz-López, Rossana Henriques

**Affiliations:** ^1^Centre for Research in Agricultural Genomics, CSIC-IRTA-UAB-UB, Barcelona, Spain; ^2^School of Biological, Earth and Environmental Sciences, University College Cork, Cork, Ireland; ^3^Environmental Research Institute, University College Cork, Cork, Ireland

**Keywords:** microRNAs, long non-coding RNAs, light, circadian clock, photoperiod

## Abstract

The biological relevance of non-protein coding RNAs in the regulation of critical plant processes has been firmly established in recent years. This has been mostly achieved with the discovery and functional characterization of small non-coding RNAs, such as small interfering RNAs and microRNAs (miRNAs). However, recent next-generation sequencing techniques have widened our view of the non-coding RNA world, which now includes long non-coding RNAs (lncRNAs). Small and lncRNAs seem to diverge in their biogenesis and mode of action, but growing evidence highlights their relevance in developmental processes and in responses to particular environmental conditions. Light can affect *MIRNA* gene transcription, miRNA biogenesis, and RNA-induced silencing complex (RISC) activity, thus controlling not only miRNA accumulation but also their biological function. In addition, miRNAs can mediate several light-regulated processes. In the lncRNA world, few reports are available, but they already indicate a role in the regulation of photomorphogenesis, cotyledon greening, and photoperiod-regulated flowering. In this review, we will discuss how light controls *MIRNA* gene expression and the accumulation of their mature forms, with a particular emphasis on those miRNAs that respond to different light qualities and are conserved among species. We will also address the role of small non-coding RNAs, particularly miRNAs, and lncRNAs in the regulation of light-dependent pathways. We will mainly focus on the recent progress done in understanding the interconnection between these non-coding RNAs and photomorphogenesis, circadian clock function, and photoperiod-dependent flowering.

## Introduction

Light is fundamental for plant life. It not only provides the energy necessary for photosynthesis but also functions as an indicator of time and place. Light quality and duration inform a plant about its neighbors in a process described as shade avoidance, which ultimately shapes its architecture and development. Light cues also function as indicators of time of the day and season [e.g., short days (SDs) in autumn and winter versus long days (LDs) in spring and summer]. Last but not least, light, together with temperature, is an input to the circadian clock, providing circadian entrainment and contributing to maintain rhythmicity and robustness of the clock. Proper clock resetting is critical for the adequate timing of biological processes, which will then reflect in plant fitness.

In parallel with seasonal and positional information, light also provides important developmental cues. For instance, during photomorphogenesis, when the young seedling emerges through the soil reaching for light, hypocotyl elongation is inhibited, while roots grow, cotyledons open and expand, and the first true leaves develop (Leivar and Monte, 2014). These morphological changes are accompanied by the development of chloroplasts and chlorophyll accumulation. Later in development, light will also regulate the time of flowering. Depending on each species requirements, specific photoperiods (the relative duration of the daily light and dark periods) will provide molecular cues, such as the accumulation of specific regulators, required for the transition of shoot apical meristems into inflorescence meristems (Andrés and Coupland, 2012).

Therefore, considering all light-dependent and/or light-regulated processes, it is not surprising that light signals promote massive transcriptional changes affecting both protein coding genes as well as non-protein coding transcripts (López-Juez et al., 2008; Wang H. et al., 2014). Although light regulation of (protein-coding) gene expression has been studied in extreme detail, the role of light in the regulation of non-protein coding RNAs is less known.

**FIGURE 1:**
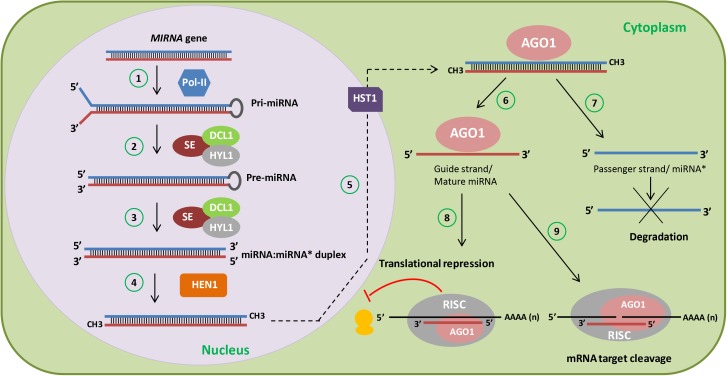
miRNA biogenesis in plants. Cartoon depicting a simplified scheme of miRNA biogenesis in plants. *MIRNA* genes are first transcribed by RNA polymerase II (Pol-II) into primary-miRNAs (pri-miRNAs) (1). Then, the dicing complex involving DICER-LIKE1 (DCL1), HYPONASTIC LEAVES1 (HYL1), and SERRATE (SE) generate the precursor-miRNA (pre-miRNA) (2) after capping, polyadenylation, and splicing. Subsequent cleavage of the pre-miRNA gives rise to the miRNA:miRNA^∗^ duplex (3). Afterward, HUA ENHANCER 1 (HEN1) stabilizes the miRNA:miRNA^∗^ duplex through 3′ methylation (4) and HASTY1 (HST1) (5) exports it to the cytoplasm. During ARGONAUTE1 (AGO1) loading, one strand is selected (guide strand or mature miRNA) (6) and the other strand (passenger strand or miRNA^∗^) is degraded (7). The miRNA strand is then loaded into the RNA-induced silencing complex (RISC) containing AGO1, which recognizes transcripts with complementarity to the miRNA and represses the translation (8) or cleaves (9) these miRNA targets.

Non-coding RNAs have been divided into different functional groups based on the size of their active forms. Non-coding RNAs shorter than 200 base pairs (bp) fall within the category of small non-coding RNAs, whereas longer ones constitute long non-coding RNAs (lncRNAs). Historically, small non-coding RNAs were the first identified and characterized in great detail. Within this group, microRNAs (miRNAs) are transcribed from *MIRNA* genes as longer precursors (primary miRNAs, pri-miRNAs) by RNA polymerase II. Plant pri-miRNAs fold into stem-loop structures and are processed by a complex containing DICER-LIKE1 (DCL1), HYPONASTIC LEAVES1 (HYL1), and SERRATE (SE), which will first release the stem-loop (pre-miRNA) structure, and then cleave it giving rise to a mature miRNA:miRNA^∗^ duplex, normally 20–22 nucleotides (nt) long, with the rarer cases of 23–25 nt, with 2-nt 3′ overhangs (Voinnet, 2009; Borges and Martienssen, 2015; Yu et al., 2017). This duplex is methylated at its 3′ ends by the methyltransferase HUA ENHANCER1 (HEN1) and this methylation is necessary to stabilize miRNAs (Yu et al., 2005). The mature miRNA strand is then selectively bound by ARGONAUTE1 (AGO1) and loaded into the RNA-induced silencing complex (RISC), which recognizes transcripts with full or partial complementarity to the miRNA and cleaves and/or represses translation of these miRNA target transcripts, therefore silencing them (Voinnet, 2009; Yu et al., 2017) (**Figure 1**). miRNAs act in particular developmental processes and/or in response to certain environmental cues, sometimes in specific tissues where they accumulate at lower levels than their targets, protein-coding transcripts.

lncRNAs, on the other hand, can exert their biological function without further processing and accumulate to similar levels as miRNAs (Liu et al., 2012). lncRNAs are classified according to their genomic location into intergenic (expressed in between coding regions), intronic (expressed from introns), or natural antisense transcripts (NATs, expressed from the complementary strand to a protein-coding gene or another lncRNA; Liu et al., 2012; Wang H. et al., 2014). Comprehensive screening approaches have revealed thousands of lncRNAs that display spatial-, temporal-, and developmental-specific patterns of expression. Interestingly, among the few lncRNAs whose biological function is known, two are involved in light-regulated processes. *HIDDEN TREASURE 1* (*HID1*) is involved in photomorphogenesis and seedling greening (Wang Y. et al., 2014; Wang et al., 2018), whereas *CDF5 LONG NON-CODING RNA* (*FLORE*) mediates the circadian regulation of flowering time (Henriques et al., 2017).

Here, we review the role of miRNAs and lncRNAs associated with, or regulated by light-dependent pathways. We will discuss light regulation of miRNA expression, biogenesis, processing, and function, as well as their role in light-related processes, such as photomorphogenesis and photoperiod-dependent flowering. We will also discuss the available reports characterizing lncRNA function in light-dependent pathways.

## MicroRNAs

Light regulation of plant miRNAs can occur through at least three different mechanisms: regulation of *MIRNA* gene transcription and its levels, regulation of miRNA processing *via* effects on components of the miRNA biogenesis machinery, and regulation of miRNA function through control of RISC factors. In turn, miRNAs can target genes encoding components of the light signaling pathways, thereby affecting light responses such as photomorphogenesis and photoperiod-dependent flowering. Below we discuss the recent progresses made in these fields of research. Due to the interconnection between light and the circadian clock, we will also discuss the available reports on circadian-regulated ncRNAs.

### Light Regulation of miRNA Levels

Light, photoperiod, and the circadian clock modulate the expression levels of *MIRNA* genes. This regulation is achieved by the presence of light-responsive elements in their promoters, and can be triggered by specific light qualities (red, blue, far-red, UV-A, and UV-B). To identify the miRNA families under this control, several screening approaches have been developed, resulting in extensive listings of light-responsive miRNAs.

For instance, the comparison of miRNAs between wild-type rice (*Oryza sativa*) plants and a *phytochrome B* (*phyB*) mutant identified 135 differentially expressed miRNAs, of which 97 were upregulated and 38 were downregulated. These miRNAs include conserved and novel miRNAs, with sizes varying between 21-nt and 24-nt, such as miR156, miR166, miR171, and miR408 (Sun et al., 2015). Differently from a previous report showing that miR172 is regulated by PHYB in potato (Martin et al., 2009), Sun et al. (2015) did not find any miR172 family member that responded to PHYB in rice, suggesting that PHYB-mediated regulation of miRNAs may differ between plant species. Using degradome sequencing, it was found that 70 rice genes were targeted by 32 differentially expressed miRNAs between the wild-type and the *phyB* mutant (Sun et al., 2015). A large proportion of them (42%) encode transcription factors, suggesting that regulation of gene expression by miRNA target genes may play an important role in PHYB-mediated light signaling. Members of the transcription factor families identified by Sun et al. (2015) are involved in light signaling in *Arabidopsis*. It is worth noting that one of the identified miRNA target genes is a homolog of the *Arabidopsis* circadian-regulated *TANDEM ZINC KNUCKLE PROTEIN* (*TZP*) gene, which is involved in blue light-associated growth (Loudet et al., 2008). However, further research is required to fully understand the role of these rice miRNAs and target genes in light-regulated processes (Sun et al., 2015).

Moreover, ELONGATED HYPOCOTYL 5 (HY5), a major regulator of photomorphogenesis that acts downstream of several photoreceptors, was shown to bind to the promoters and regulate the expression of several *MIRNA* genes, such as *MIR156D, MIR402, MIR408*, and *MIR858A* in *Arabidopsis* (Zhang et al., 2011). This, together with the effect of PHYB on miRNA levels (Sun et al., 2015), strongly suggests that light affects miRNA abundance. In support of this, small RNA sequencing indicates that the miRNA population of the seedling apical hook changes after treating dark-grown soybean (*Glycine max*) seedlings with far-red light. The levels of several miRNAs (miR166, miR167, miR168, miR394, miR396, miR530, miR1507, miR1508, miR1509, and miR2218) respond to far-red light in different parts of the soybean seedling, in most cases showing upregulation by far-red light (Li et al., 2014). Therefore, light seems to upregulate miRNAs in the apical hook, leading to downregulation of target genes that presumably repress apical hook and cotyledon opening.

Light has strong effects on plant metabolism, especially in the regulation of fundamental plant processes such as photosynthesis (Kooke and Keurentjes, 2012). To understand the involvement of miRNAs in light regulation of potato metabolism, Qiao et al. (2017) used high-throughput sequencing to study the expression levels of miRNAs and mRNAs in dark-treated and red-light-treated leaves and detached tuber skin in potato. Among the 69 known and novel miRNAs that were differentially regulated, most were upregulated in leaves, whereas in tuber skin, about half of them were upregulated and half downregulated (Qiao et al., 2017). Notably, the miR399 family and the novel miRNA miRn55 showed opposite light responses in leaves (upregulation) and tuber skin (downregulation). General gene expression analyses revealed that most differentially expressed mRNAs (67%) were upregulated in tuber skin, whereas in leaves, about half were upregulated and half downregulated. Only 14% of differentially expressed miRNAs and 12% of differentially expressed genes were common to leaves and tuber skin, indicating that the molecular response to red light differs between these tissues. Nevertheless, there was an effect of light on genes involved in primary and secondary metabolism in both tissues, especially in carbohydrate metabolism, which is downregulated, and alkaloid metabolism, which is upregulated. Repression of carbohydrate metabolism was supported by anti-correlation between miRNAs and mRNAs, which suggests downregulation of putative miRNA targets involved in this process (Qiao et al., 2017). Although confirmation of these findings using additional methods is required, this work points to a role of miRNAs in the regulation of metabolic pathways affected by red light in potato.

Additional support to the effect of red light on miRNA levels has been obtained in *Arabidopsis*. In this species, the most differentially expressed miRNAs in red light versus darkness are miR160, miR163, miR319, miR394, miR779, miR851, miR854, and miR2111 (Sun et al., 2018). At least part of this effect is mediated by PHYTOCHROME-INTERACTING FACTOR4 (PIF4), a transcription factor that is a negative regulator of PHYB-mediated red light signaling. In a *pif4* mutant, 22 mature miRNAs, representing 16 miRNA families, show altered levels relative to wild-type plants under red light. PIF4 promotes the expression of genes encoding miR156/157, miR160, miR165/166, miR167, miR170/171, and miR394 and represses the expression of genes encoding miR172 and miR319 by binding to the promoters of several of these genes. In addition to acting as a transcription factor for *MIRNA* genes, PIF4 regulates miRNA processing, as discussed below (Sun et al., 2018).

Besides light exposure, photoperiod can also control miRNA accumulation. Li et al. (2015) used high-throughput sequencing to identify miRNAs regulated by photoperiod in soybean. Comparing miRNA levels in seedlings grown under LDs and SDs revealed 37 miRNA families to be day-length-responsive. Five members of the miR156 family were induced under LD conditions and this correlated with downregulation of four miR156-target *SQUAMOSA PROMOTER-BINDING PROTEIN-LIKE* (*SPL*) genes. Given that miR156 overexpression delays flowering in soybean (Cao et al., 2015), lower miR156 levels under SD than LD may explain the fact that soybean flowering is induced by SD. Conversely, the expression of six members of the miR172 family was induced under SD conditions and this was coordinated with the repression of ten predicted targets belonging to the *APETALA2* (*AP2*)-like family (Li et al., 2015). In addition to miR156 and miR172, other conserved miRNAs, such as miR159/319, miR166, miR167, miR395, and miR408 were also shown to be photoperiod regulated in soybean.

The simplified model of the circadian clock places light as an input that confers clock entrainment. Similarly to light, the clock also controls the expression of a vast set of genes, especially at the dark/light transition (Michael et al., 2008), thus allowing plants to anticipate certain daily processes. Interestingly, the circadian clock can also regulate the expression of protein-coding as well as non-coding transcripts (Hazen et al., 2009). To uncover circadian-regulated miRNAs, Siré et al. (2009) compared mature and pri-miRNA transcript levels under light/dark cycles or under free-running (continuous light) conditions for two consecutive days. It was found that miR167, miR168, miR171, and miR398 levels oscillated under light/dark cycles, peaking around the light/dark transition. However, for certain miRNAs, the waving pattern was not accompanied by a clear antiphasic expression of their targets but rather by a phase delay. This was the case of miR171/*SCARECROW-LIKE 6* (*SCL6*, also named *SCL6-IV, HAM3*, and *LOM3*) and miR398/*Cu/Zn SUPEROXIDE DISMUTASE 2* (*CSD2*) pairs, where target gene expression is reduced as the miRNA levels increase. However, in the case of the miR168/*AGO1* and miR167/*AUXIN RESPONSE FACTOR 6* (*ARF6*) pairs, an in-phase pattern of accumulation was found, suggesting that miRNAs could regulate their targets by incorporating feedback loops. However, when these modules were tested under free-running conditions, a clear oscillation was not found, an indication that these miRNAs are most likely light regulated.

In another report, Li et al. (2016) addressed the mechanism by which the circadian clock regulates carbon and nitrogen metabolism in rice plants grown in field conditions. Using a comprehensive approach, the authors investigated how light/dark cycles control certain biological processes. They investigated metabolic and enzymatic activities, as well as gene expression and alternative splicing events that could show rhythmic behavior. In their search for oscillatory miRNAs associated with carbon and/or nitrogen metabolism, Li et al. (2016) uncovered miR1440b, miR1877, miR2876-5p, and miR5799 as possible rice miRNA candidates. These miRNAs oscillate under light/dark cycles and negatively correlate with their targets at the majority of time points analyzed. However, it still remains to be seen whether these are *bona fide* circadian miRNAs oscillating under free-running conditions.

#### UV-Responsive miRNAs

Since different light qualities can trigger diverse developmental responses, some of which can be detrimental, several screening approaches have been designed to understand how light, especially high light, UV-A, and UV-B radiation can modulate accumulation of miRNAs and their targets. Zhou et al. (2016), using seedlings from *Brassica rapa* exposed to blue light and UV-A, identified 15 conserved and 226 novel differentially expressed miRNAs that could target genes encoding regulators of plant growth, development, and photomorphogenesis (Zhou et al., 2016). Blue light and UV-A exposure slightly downregulated miR156/157 expression, which correlated with upregulation of their targets, *SPL9* (Bra004674) and *SPL15* (Bra003305), which encode transcription factors involved in the regulation of many processes, including the juvenile-to-adult transition, flowering, and secondary metabolism, especially anthocyanin biosynthesis (Wu et al., 2009; Gou et al., 2011; Wang and Wang, 2015). In *Arabidopsis*, miR156 and SPLs are involved in complex feedback loops (Wu et al., 2009; Yant et al., 2010). As plants age, miR156 levels decrease and *SPL*s (e.g., *SPL9*) accumulate, inhibiting anthocyanin biosynthesis (Gou et al., 2011). Zhou et al. (2016) hypothesize that blue and UV-A light activate genes involved in light signaling and some of them promote anthocyanin biosynthesis. When anthocyanin levels increase, then the regulatory feedback loops involving miR156/157 would act to balance its metabolism.

Similar to UV-A, UV-B radiation affects plant growth, physiology, and metabolism (Dotto and Casati, 2017). In one of the first reports on the effect of light on miRNAs, Zhou et al. (2007) used a computational approach to predict *MIRNA* genes induced by UV-B light in *Arabidopsis*. miRNAs belonging to 11 miRNA families were identified as putatively upregulated by UV-B light (Zhou et al., 2007). Although these results have not been experimentally confirmed in *Arabidopsis*, several reports have shown the effect of UV-B on miRNA levels in aspen (*Populus tremula*), maize (*Zea mays*), and wheat (*Triticum aestivum*). Jia et al. (2009) found that the level of eight miRNA families increased and that of seven families decreased in response to UV-B light stress in aspen. In maize, from the 16 UV-B-regulated miRNA families detected, 6 were upregulated and 10 downregulated (Casati, 2013). UV-B-treated wheat plants showed upregulation of three and downregulation of another three known miRNAs, in addition to a novel wheat miRNA, which was slightly upregulated shortly after UV-B exposure and then was downregulated (Wang et al., 2013). Thirteen UV-B-responsive miRNA families, i.e., miR156/157, miR159, miR160, miR164, miR165/166, miR167, miR169, miR170/171, miR172, miR393, miR395 miR398, and miR399, are common to at least two plant species, indicating substantial conservation in the miRNAs that respond to UV-B light. Although the direction of the effect (upregulation or downregulation) on several miRNAs differs between species, others are consistently promoted (miR165/166, miR167, and miR398) or repressed (miR395) by UV-B light in three species. This conservation probably reflects important roles of these miRNAs in plant responses to UV-B radiation.

The upstream regulatory regions of the UV-B-regulated *MIRNA* genes contain numerous light-related motifs, such as GT-1 sites, G boxes, I-boxes, CCAAT-boxes, and GATA-boxes (Zhou et al., 2007; Jia et al., 2009; Wang et al., 2013), consistent with the effect of light on these genes. Stress-responsive *cis*-elements were also found in several of those promoters (Jia et al., 2009; Wang et al., 2013). Given that some of these miRNAs are involved in responses to abiotic and biotic stresses (Casati, 2013; Li et al., 2017), we hypothesize that these miRNAs might coordinate adaptive responses to diverse threats.

Among the targets of the UV-B-regulated miRNAs are transcription factors, several factors involved in auxin signaling and factors involved in responses to other stresses (Zhou et al., 2007; Jia et al., 2009; Casati, 2013). In most cases, as would be expected, there is an inverse correlation between the effect of UV-B on miRNAs and on their corresponding target transcripts (Zhou et al., 2007; Jia et al., 2009; Casati, 2013; Wang et al., 2013). For instance, in maize, an increase in miR165/166 correlated with inhibition of their target gene, encoding a HD-ZIP III transcription factor, *ROLLED LEAF1* (*RLD1*; Casati, 2013). In agreement with the role of these miRNAs in delimitating *HD-ZIP III* transcripts to the adaxial side of leaf primordia (Juarez et al., 2004), UV-B-dependent repression of *RLD1* was associated with leaf arching, suggesting a role for miR165/166 in this UV-B response (Casati et al., 2006). In aspen and wheat, miR395 downregulation is associated with upregulation of its targets encoding ATP sulfurylases (APS), enzymes involved in inorganic sulfate assimilation (Jia et al., 2009; Wang et al., 2013). miR395 and *APS* are inversely regulated by sulfate starvation, and miR395 overexpression reduces *APS* transcript levels and affects the response of *Arabidopsis* to abiotic stress conditions (Jones-Rhoades and Bartel, 2004; Kim et al., 2010), linking again a UV-B-regulated miRNA with other stress responses.

This is also the case of miR164, whose downregulation in UV-B-treated maize leaves is associated with increased levels of two stress-responsive *NAC-DOMAIN PROTEIN* target transcripts, as well as *EXOSTOSIN PROTEIN-LIKE* and an *ASPARTYL PROTEASE* (Hegedus et al., 2003; Casati, 2013). Interestingly, other miRNAs (miR171, miR172, miR156, and miR529) that target genes involved in critical developmental transitions (juvenile-to-adult transition and/or flowering) were also downregulated by UV-B light (Lauter et al., 2005; Xie et al., 2006; Chuck et al., 2008; Cuperus et al., 2011). This suggests that UV-B exposure promotes miRNA-mediated responses that delay certain developmental transitions, allowing plants either to repair UV-B-induced damage or to adapt to these stressful conditions.

Overall, the analysis of UV-B-regulated miRNAs in several plant species indicates that there is a core set of conserved UV-B-responsive miRNAs. Nevertheless, the findings also suggest that different species may recruit distinct miRNAs in order to cope with UV-B stress. Understanding the role of these miRNAs in UV-B-regulated processes awaits further investigation.

#### miRNAs Accumulated in High Light Conditions

Besides UV light, high light can also be detrimental for the development of different plant species. To address how light, especially high light, controls miRNA expression in ma bamboo (*Dendrocalamus latiflorus*) grown under LD conditions, Zhao et al. (2013) generated small RNA libraries and determined how miRNA families were regulated. Several conserved miRNAs were identified, miR168 being the most abundant family, followed by miR156/157, miR535, miR165/166, and miR167. Interestingly, miR172 was not found, probably due to the unique flowering cycle of ma bamboo. Using stem-loop RT-qPCR, Zhao et al. (2013) determined the level of novel miRNAs in plants grown under regular white light, high light, or dark. However, the role of these miRNAs in high-light-mediated processes still remains to be determined.

A global analysis of the light-regulated miRNAs reported so far reveals that numerous miRNA families are affected by diverse light conditions (**Table 1**). Some of them, such as miR156/157, miR159/319, miR164, miR165/miR166, miR167, miR170/171, miR172, miR395, miR398, miR399, and miR408, have been identified as responsive to several light conditions in at least five plant species, strongly suggesting that they may play roles in light responses (**Table 1**). However, light could also modulate miRNA processing and/or activity toward its targets. Below we present the most recent reports addressing this type of regulation.

**Table 1:** miRNA families differentially regulated by light.

miRNA^1^ family	Light conditions^2^	Plant species^3^	Reference
miR156/157	W, FR, B, UV-A, UV-B, *phyB* mutant, *pif4* mutant, LD/SD, bagging treatment	Ath, Gma, Mdo, Osa, Ppy, Ptr, Stu, Tae, Zma	Zhou et al., 2007, 2016; Jia et al., 2009; Casati, 2013; Wang et al., 2013; Bhogale et al., 2014; Tsai et al., 2014; Li et al., 2015; Sun et al., 2015, 2018; Qu et al., 2016; Lin et al., 2017; Qian et al., 2017; Xie et al., 2017
miR167	W, FR, UV-B, *phyB* mutant, *pif4* mutant, L/D cycles, LD/SD, bagging treatment	Ath, Gma, Mdo, Osa, Ptr, Tae	Zhou et al., 2007; Jia et al., 2009; Siré et al., 2009; Wang et al., 2013; Li et al., 2014, 2015; Sun et al., 2015, 2018; Qu et al., 2016; Lin et al., 2017
miR170/171	W, UV-B, *phyB* mutant, *pif4* mutant, L/D cycles, LD/SD, bagging treatment	Ath, Gma, Mdo, Osa, Tae, Zma	Zhou et al., 2007; Siré et al., 2009; Casati, 2013; Wang et al., 2013; Fan et al., 2015; Li et al., 2015; Sun et al., 2015, 2018; Qu et al., 2016; Lin et al., 2017
miR165/166	W, FR, R, UV-B, *phyB* mutant, *pif4* mutant, LD/SD	Ath, Gma, Osa, Ptr, Stu, Zma	Zhou et al., 2007; Jia et al., 2009; Casati, 2013; Li et al., 2014, 2015; Sun et al., 2015, 2018; Lin et al., 2017; Qiao et al., 2017
miR398	W, R, UV-A, UV-B, L/D cycles, LD/SD, bagging treatment	Ath, Gma, Mdo, Ptr, Stu, Zma	Zhou et al., 2007, 2016; Jia et al., 2009; Siré et al., 2009; Casati, 2013; Li et al., 2015; Qu et al., 2016; Lin et al., 2017; Qiao et al., 2017
miR159/319	W, R, UV-B, *phyB* mutant, *pif4* mutant, LD/SD,	Ath, Gma, Osa, Ptr, Tae	Zhou et al., 2007; Jia et al., 2009; Wang et al., 2013; Tsai et al., 2014; Li et al., 2015; Sun et al., 2015; Lin et al., 2017; Sun et al., 2018
miR172	R, B, UV-B, *pif4* mutant, LD/SD, bagging treatment	Ath, Mdo, Gma, Stu, Zma	Jung et al., 2007; Zhou et al., 2007; Martin et al., 2009; Casati, 2013; Li et al., 2015; Qu et al., 2016; Sun et al., 2018
miR396	W, FR, UV-A, UV-B, *phyB* mutant, LD/SD	Ath, Gma, Osa, Zma	Casadevall et al., 2013; Casati, 2013; Li et al., 2014, 2015; Sun et al., 2015; Zhou et al., 2016; Lin et al., 2017
miR408	W, R, UV-B, *phyB* mutant, LD/SD, bagging treatment	Ath, Gma, Mdo, Osa, Ptr, Stu	Jia et al., 2009; Zhang et al., 2014; Li et al., 2015; Sun et al., 2015; Qu et al., 2016; Lin et al., 2017; Qiao et al., 2017
miR160	W, R, UV-B, *phyB* mutant, *pif4* mutant, bagging treatment	Ath, Mdo, Osa, Ptr	Zhou et al., 2007; Jia et al., 2009; Sun et al., 2015, 2018; Qu et al., 2016; Lin et al., 2017
miR169	W, UV-B, *phyB* mutant, *pif4* mutant, LD/SD	Ath, Gma, Osa, Ptr	Zhou et al., 2007; Jia et al., 2009; Li et al., 2015; Sun et al., 2015, 2018; Lin et al., 2017
miR164	UV-B, *phyB* mutant, bagging treatment	Mdo, Ptr, Tae, Osa, Zma	Jia et al., 2009; Casati, 2013; Wang et al., 2013; Sun et al., 2015; Qu et al., 2016
miR395	W, UV-B, bagging treatment	Ath, Mdo, Ptr, Tae, Zma	Jia et al., 2009; Casati, 2013; Wang et al., 2013; Qu et al., 2016; Lin et al., 2017
miR399	W, R, UV-B, bagging treatment	Ath, Mdo, Ptr, Stu, Zma	Jia et al., 2009; Casati, 2013; Qu et al., 2016; Lin et al., 2017; Qiao et al., 2017
miR168	FR, UV-B, L/D cycles, LD/SD	Ath, Gma, Ptr	Jia et al., 2009; Siré et al., 2009; Li et al., 2014, 2015
miR393	W, UV-B, bagging treatment	Ath, Mdo, Ptr	Zhou et al., 2007; Jia et al., 2009; Qu et al., 2016; Lin et al., 2017
miR858	W, bagging treatment	Ath, Mdo	Qu et al., 2016; Sharma et al., 2016; Wang et al., 2016; Lin et al., 2017
miR1511	B, *phyB* mutant, LD/SD, bagging treatment	Ath, Gma, Mdo, Osa	Li et al., 2015; Sun et al., 2015; Qu et al., 2016; Zhou et al., 2016
miR163	W, R, B	Ath	Chung et al., 2016; Lin et al., 2017; Sun et al., 2018
miR390	W, UV-B, LD/SD	Ath, Gma, Ptr	Jia et al., 2009; Li et al., 2015; Lin et al., 2017
miR482	R, LD/SD, bagging treatment	Gma, Mdo, Stu	Li et al., 2015; Qu et al., 2016; Qiao et al., 2017
miR391	B, UV-A, bagging treatment	Ath, Mdo	Qu et al., 2016; Zhou et al., 2016
miR394	FR, R, *pif4* mutant	Ath, Gma	Li et al., 2014; Sun et al., 2018
miR397	R, *pif4* mutant	Ath, Stu	Qiao et al., 2017; Sun et al., 2018
miR403	LD/SD, bagging treatment	Gma, Mdo	Li et al., 2015; Qu et al., 2016
miR444	UV-B, *phyB* mutant	Osa, Zma	Casati, 2013; Sun et al., 2015
miR903			
miR472	R, UV-B, *pif4* mutant	Ath, Ptr	Jia et al., 2009; Sun et al., 2018
miR477	R, bagging treatment	Mdo, Stu	Qu et al., 2016; Qiao et al., 2017
miR530	FR, *phyB* mutant	Gma, Osa	Li et al., 2014; Sun et al., 2015
miR535	*phyB* mutant, bagging treatment	Mdo, Osa	Sun et al., 2015; Qu et al., 2016
miR827	R, *phyB* mutant	Osa, Stu	Sun et al., 2015; Qiao et al., 2017
miR833	W, *pif4* mutant	Ath	Lin et al., 2017; Sun et al., 2018
miR1507	FR, LD/SD	Gma	Li et al., 2014, 2015
miR1508			
miR2111	R, B, UV-A	Ath	Zhou et al., 2016; Sun et al., 2018
miR2876	*phyB* mutant, L/D cycles	Osa	Sun et al., 2015; Li et al., 2016
miR5072	B, *phyB* mutant	Ath, Osa	Sun et al., 2015; Zhou et al., 2016
miR5139			
miR5368	LD/SD, *phyB* mutant	Gma, Osa	Li et al., 2015; Sun et al., 2015


*In this table, we present a summary of the different miRNAs that respond to two or more light treatments. ^1^miRNA families reported to be regulated by light in at least two papers have been included. miRNA families are ordered according to the number of papers reporting their light regulation. ^2^W, white light; FR, far-red light; R, red light; B, blue light; L/D, light/dark; LD/SD, long days/short days. ^3^Ath, Arabidopsis thaliana; Gma, Glycine max (soybean); Mdo, Malus x domestica (apple); Osa, Oryza sativa (rice); Ppy, Pyrus pyrifolia (Chinese sand pear); Ptr, Populus tremula (aspen); Stu, Solanum tuberosum (potato); Tae, Triticum aestivum (wheat); Zma, Zea mays (maize).*

### Regulation of miRNA Biogenesis, Processing, and Function by Light

**FIGURE 2:**
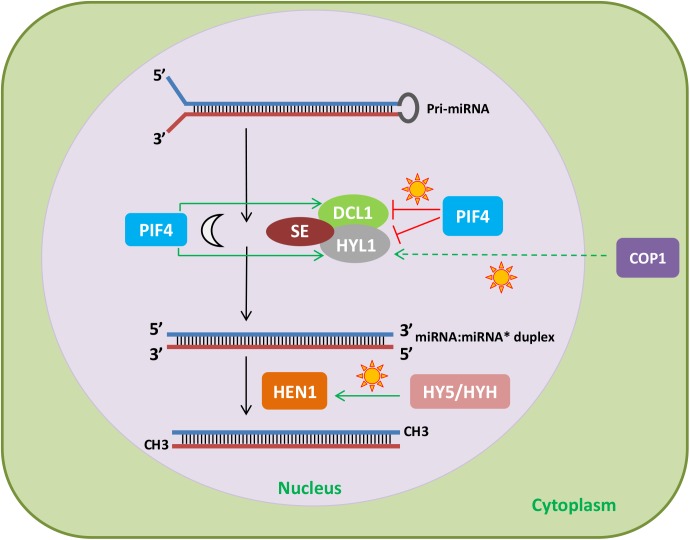
Effect of light signaling components on the miRNA biogenesis and processing machinery. The light signaling factors COP1 and PIF4 regulate the stability of HYL1. In the light, COP1, probably through degradation of an unknown protease, increases HYL1 protein levels. In addition, PIF4 stabilizes HYL1 and DCL1 in the dark and destabilizes them under red light. Finally, HY5 and HYH promote *HEN1* expression in response to light. Therefore, light can affect miRNA levels by regulating light signaling components that modulate the abundance of miRNA biogenesis and processing factors.

Besides regulating *MIRNA* gene expression, light can also modulate the levels and the activity of mature miRNAs. This can be achieved by a direct connection between light and regulators of the miRNA biogenesis pathway. HYL1 is a RNA-binding protein involved in miRNA processing (Yu et al., 2017). In *Arabidopsis*, HYL1 protein levels are regulated by CONSTITUTIVE PHOTOMORPHOGENIC 1 (COP1), an E3 ubiquitin ligase that mediates the proteasomal degradation of light signaling factors, such as photoreceptors (Cho et al., 2014). It was found that the reduction of miRNA levels in *cop1* mutants relative to wild-type plants greatly overlapped with that observed in the *hyl1* mutant. Further analyses showed that COP1 regulates HYL1 protein levels but not through direct ubiquitination. Combining protein synthesis blockers with either autophagy or 26S proteasome inhibitors revealed that HYL1 was degraded by an unknown protease, which removed its N-terminal fragment, where two RNA-binding domains, essential for miRNA processing, are located (Cho et al., 2014). Light regulates COP1 nucleocytoplasmic shuttling (Jang et al., 2010) and, therefore, during the day, light stabilizes HYL1 due to cytoplasmic accumulation of COP1, which would target the protease acting on HYL1 degradation (**Figure 2**). This regulation could control miRNA processing and thus help understand the light/dark accumulation pattern of several mature miRNAs, such as miR167, miR168, miR171, and miR398, previously described by Siré et al. (2009).

HYPONASTIC LEAVES1 protein levels are also controlled by PIF4, another protein involved in photomorphogenesis (Sun et al., 2018). Both HYL1 and DCL1 interact with PIF4, which destabilizes them under red light and stabilizes them in the dark. The mechanism by which PIF4 controls the levels of HYL1 and DCL1 is unknown, but it does not involve transcriptional regulation or the ubiquitin-proteasome pathway. Given that PIF4 interacts with the photoreceptor PHYB and the miRNA processing factors DCL1 and HYL1, the question arises as to whether DCL1 and HYL1 affect light responses (**Figure 2**). Indeed, *dcl1* and *hyl1* mutants show shorter hypocotyls than wild-type plants under red light, indicating that DCL1 and HYL1 play negative roles in photomorphogenesis (Sun et al., 2018).

Another miRNA processing factor regulated by light signaling pathways is HEN1. Light induction of *HEN1* expression results from the coordinated action of HY5 and HY5 homolog (HYH; **Figure 2**), which positively regulate *HEN1* downstream of several photoreceptors (Tsai et al., 2014). Consistent with this regulation, HEN1 and HYL1 are negative regulators of photomorphogenesis, in the case of HEN1, this is due to its role as a repressor of key transcription factors of this process (Tsai et al., 2014). In addition, alternative splicing of transcripts encoding several miRNA biogenesis and processing factors, such as DCL1, HYL1, and HEN1, is affected by different light qualities, adding a new layer to the regulation of miRNAs by light (reviewed in Hernando et al., 2017).

The de-etiolation process can also affect miRNA activity toward its targets. Lin et al. (2017) matched small RNA profiling with degradome sequencing in *Arabidopsis* and found that, although light exposure did not change dramatically the level of most miRNAs, it could modulate their target cleavage activity. In fact, with the exception of miR163, most miRNAs showed little fluctuations in their amount upon light transition. This did not correspond with the analysis of degradome signatures, implying that de-etiolation especially promoted the degradation of miR156/157 and miR396 targets, and thus suggesting higher cleavage activity for these families (Lin et al., 2017). However, although Lin et al. (2017) report that miR168 regulates *AGO1* levels under light and could modulate RISC activity and the efficiency of miRNA target cleavage, the mechanisms underlying this regulation by light still need to be demonstrated.

Interestingly, another report has shown AGO1 involvement in light regulation of adventitious rooting and hypocotyl elongation. *Arabidopsis ago1* mutants are hypersensitive to light, probably due to deregulation of the PHYA-dependent light signal transduction pathway (Sorin et al., 2005). Moreover, de-etiolation of an *Arabidopsis* hypomorphic *ago1-27* mutant in far-red light is impaired, indicating that normal photomorphogenesis requires AGO1 (Li et al., 2014). Given that AGO1 is an essential component of the RISC, these findings suggest that miRNA function is involved in far-red light responses.

Therefore, several components of the miRNA machinery are involved in certain light responses. On the other hand, light modulates *MIRNA* gene expression, accumulation of mature miRNAs by regulating components of the biogenesis pathway, as well as miRNA activity. How miRNAs participate in light as well as photoperiod signaling events will be discussed next.

### miRNAs Involved in Light and Photoperiod Signaling

Our analysis revealed several miRNA families that accumulate upon certain light treatments in different species. Most of these miRNAs mediate critical biological processes necessary for plants to adjust to these changes. They can regulate metabolism, plant development, and certain responses to stress.

#### miR396 and UV-B Regulation of Cell Proliferation

For instance, UV-B radiation affects cell proliferation in several species (Dotto and Casati, 2017). In *Arabidopsis*, UV-B light upregulates miR396 levels, leading to repression of the miR396 target genes *GROWTH REGULATING FACTOR1* (*GRF1*), *GRF2*, and *GRF3*, with consequent inhibition of cell proliferation in leaves (Casadevall et al., 2013). In fact, either reducing miR396 activity or expressing miR396-resistant forms of *GRF3* or *GRF2* results in reduced sensitivity of leaf growth to UV-B irradiation, confirming that *GRF*s mediate this phenotype. Nevertheless, downregulation of GRF3 by UV-B light also seems to occur through miR396-independent mechanisms. In addition, miR396-mediated repression of leaf cell proliferation by UV-B radiation is dependent on the mitogen-activated protein kinase MPK3, known to be involved in the response to UV-B stress (Casadevall et al., 2013). Interestingly, in UV-B-treated maize leaves, miR396 inhibition had the opposite effect on *GRF*s, which were upregulated (Casati, 2013). This could be due to the different developmental stages analyzed in *Arabidopsis* and maize, although further studies are necessary to clarify the role of miR396 in the regulation of UV-B-dependent responses in dicots and monocots.

#### miRNAs Regulating Metabolism

As mentioned before, light controls many metabolic pathways in plants. Within this context, light-regulated miRNAs were shown to modulate metabolic events, such as methylation of specific signaling molecules (e.g., hormones), nutrient allocation, and pigment synthesis/accumulation.

miR163 as well as *pri-miR163* were recently shown to be highly induced by light in *Arabidopsis* (Chung et al., 2016; Lin et al., 2017). Moreover, miR163 may target *PXMT1* (At1g66700), a neighbor gene encoding a putative 1,7-paraxanthine methyltransferase that has been implicated in the methylation of natural chemicals such as hormones (Chung et al., 2016). Whereas *pri-miR163* is induced in red, blue, and white light, and *PXMT1* expression is inhibited upon light exposure, light does not affect *pri-miR163* maturation and processing. Both *pri-miR163* and its target *PXMT1* accumulate in roots, where miR163 accumulation increases root length, especially in the elongation/differentiation zone, most likely by inhibiting *PXMT1*. Interestingly, the miR163/*PXMT1* pair also seems to regulate germination, probably by controlling the early stages of plant development since radicle emergence to seedling de-etiolation. However, to fully understand the role of miR163 in this process, a more extensive functional characterization of *PXMT1* would be necessary.

Plant metabolism requires proper nutrient allocation, especially in the case of nutrients that constitute co-factors and are present in different proteins and biological processes. This is the case of copper, since it is required for electron transport in photosynthesis, defense against reactive-oxygen species as well as ethylene perception (Zhang et al., 2014). Maintenance of copper homeostasis is critical to photosynthesis efficiency and thus plant growth. This is achieved by the SPL7 transcription factor, which functions as a copper sensor able to adjust target gene expression upon changing copper levels. Surprisingly, light and copper seem to regulate gene expression of many shared biological processes, suggesting a direct connection between the transcriptional regulators SPL7 and HY5 (Zhang et al., 2014). Using chromatin immunoprecipitation and RNA sequencing, Zhang et al. (2014) characterized the SPL7 regulon and found it to overlap with HY5. This analysis revealed *MIR408* as a SPL7/HY5 common target, with SPL7 being the predominant transcription factor determining *MIR408* levels, thus confirming previous work showing that HY5 regulates *MIR408* expression (Zhang et al., 2011). Nevertheless, HY5/SPL7 coordinated regulation allowed miR408 accumulation upon copper deficiency and high light. Confirming this regulation, *MIR408* silencing phenotypes were similar to those of the *hy5 spl7* double mutant, whereas miR408 constitutive expression partially rescued them, suggesting that miR408 is a critical component of the SPL7/HY5 network (Zhang et al., 2014). Further analysis of the expression levels of the miR408 targets, *LACCASE12* (*LAC12*) and *LAC13*, showed that the SPL7/HY5/miR408 module allows differential regulation of very closely related genes. Therefore, this signaling network constitutes a molecular mechanism that integrates light and copper signals to maintain copper homeostasis and consequently efficient photosynthetic rates that promote plant growth.

##### miRNAs and pigment synthesis

Flavonoids are a group of phenolic compounds that include flavonols, flavones, isoflavones, and anthocyanins (Sharma et al., 2016). Due to their molecular diversity, flavonoids can confer protection against biotic and abiotic stress, as well as regulate plant growth and development. Anthocyanins, for instance, include a wide range of pigments (blue, red, and purple), which can not only protect against UV radiation but can also attract pollinators (Wang et al., 2016). Due to their relevance to plant life, the biosynthesis and regulation of all these compounds have been extensively studied. The transcription factors MYB-like 2 (MYBL2) and SPL9 are negative regulators of anthocyanin biosynthesis in *Arabidopsis* (Dubos et al., 2008; Matsui et al., 2008; Gou et al., 2011). On the other hand, overexpression of miR156 increases anthocyanin levels in *Arabidopsis*, since some miR156 target genes, including *SPL9*, repress anthocyanin biosynthesis (Gou et al., 2011; Cui et al., 2014). In Chinese sand pear (*Pyrus pyrifolia*) fruits, bagging and subsequent re-exposure to light causes anthocyanin accumulation in fruit peel, giving red color to the fruits (Qian et al., 2013). Qian et al. (2017) hypothesized that miR156 and *SPL*s would also be involved in anthocyanin accumulation in pears in response to bagging treatments. They detected miR156 in peels of bagging-treated pear fruits and found that the promoters of two pear *PpMIR156* genes contained light-responsive elements. After bag removal, miR156 levels increased, whereas *PpSPL* transcripts decreased. This was accompanied by upregulation of homologs of genes encoding transcription factors that control anthocyanin biosynthesis, and followed by upregulation of anthocyanin biosynthesis genes (Qian et al., 2017). Although functional analyses of miR156 and *PpSPL* genes in pear have not yet been reported, these results point to a possible role of miR156 and PpSPL proteins in light-induced anthocyanin biosynthesis in bagging-treated pear fruits.

In a related report, Qu et al. (2016) investigated the miRNA profiling of “Granny Smith” apple peels upon re-exposure to sunlight after fruit bagging, using high-throughput sequencing of small RNA libraries. They found that over 67% of differentially expressed miRNAs were downregulated in the bagged group as compared to the unbagged control. These findings suggest the existence of a light-regulated pool of miRNAs in apple peel. miR156, miR160, miR171, miR172, miR395, and miR398 were differentially expressed upon sunlight re-exposure. Interestingly, these miRNAs families had previously been identified as UV-B-regulated miRNAs in several plant species (see Section “UV-Responsive miRNAs”). In *Arabidopsis*, in addition to miR156, miR858 positively regulates anthocyanin biosynthesis by targeting specific repressors of this process (Gou et al., 2011; Qian et al., 2013; Cui et al., 2014; Wang et al., 2016). In tomato, miR858 also promotes anthocyanin biosynthesis (Jia et al., 2015). Interestingly, miR156, miR828, and miR858 expression levels varied upon apple fruit debagging, suggesting their involvement in anthocyanin biosynthesis in apple peels. In fact, miR156 correlated with anthocyanin accumulation only in “Granny Smith,” whereas miR5072 did it only in the “Starkrimson” cultivar. These results highlight the high specificity of certain miRNA families to control particular responses, such as pigment accumulation to protect against light stress.

As previously mentioned, miR858 is a positive regulator of anthocyanin biosynthesis (Wang et al., 2016). In *Arabidopsis, MIR858A* expression is induced by light, especially high light, in a HY5-dependent manner. Upon transfer from dark to high light, *MIR858A* level oscillates, suggesting also circadian regulation. Interestingly, miR858a overexpression partially complements the long hypocotyl phenotype of the *hy5* mutant and rescues the anthocyanin levels of this mutant. miR858a and miR858b can promote anthocyanin biosynthesis in two ways: (1) by indirectly inducing the expression of genes encoding components of the anthocyanin biosynthetic pathway and (2) by inhibiting protein accumulation of their target gene encoding the repressor MYBL2 protein, most likely at the translational level. HY5 directly binds and represses the *MYBL2* promoter probably by a mechanism that involves the loss of H3K9Ac and H3K4me3-positive histone marks (Wang et al., 2016). Therefore, HY5 has a dual effect in promoting anthocyanin biosynthesis: it binds and represses *MYBL2*, and it promotes *MIR858A* expression in response to light (e.g., high light) signals.

It has been shown that *hen1* mutants, which have reduced levels of mature miRNAs, accumulate high anthocyanin levels (Tsai et al., 2014), a result somehow contradictory with miR156 and miR858 promoting anthocyanin biosynthesis. However, Sharma et al. (2016) propose a different function for miR858 in the regulation of flavonoid biosynthesis. They found that miR858 targets the positive regulators of flavonoid biosynthesis *MYB11, MYB12*, and *MYB111*. Moreover, transcriptional profiling of miR858-overexpressing lines (miR858-OX) revealed *MYB20, MYB42*, and *MYB83* as new targets of miR858. Since these transcription factors regulate several growth responses, the authors characterized growth phenotypes of miR858-OX and miR858a target mimic lines (*MIM858*), in which miR858a activity is silenced. While miR858-OX plants showed enhanced growth, early flowering, and an increase in seed size, *MIM858* plants displayed reduced growth, late flowering, and smaller seeds (Sharma et al., 2016). The metabolic profiling of these lines confirmed their opposite effect on flavonoid accumulation, since miR858 depletion resulted in the accumulation of major flavonoids, whereas they were significantly reduced in miR858-OX. This was accompanied by an opposite effect on lignin biosynthesis, since miR858-OX showed increased lignification and upregulation of lignin biosynthetic genes. These results indicate that miR858 could differently regulate anthocyanin, other flavonoids, and lignin biosynthesis. This could be achieved by targeting different MYB transcription factors in response to distinct light conditions (white light versus low or high light). This differential regulation could then help maintain a metabolic flux balance between the different biosynthetic pathways.

Chlorophylls are critical plant pigments involved in light absorption and energy transfer during photosynthesis. However, this fundamental process for plant life also generates reactive oxygen species, which are detrimental for plant growth and development. Considering that chlorophyll biosynthesis precursors are also sources for reactive oxygen species, it is of paramount importance to tightly regulate this pathway. One of such mechanisms includes miR171 and miR171-targeted *SCL* genes, also known as *HAIRY MERISTEM* (*HAM*) or *LOST MERISTEMS* (*LOM*). In *Arabidopsis*, miR171 positively regulates chlorophyll biosynthesis in the light through downregulation of miR171-targeted *SCLs*, leading to upregulation of protochlorophyllide oxidoreductase (POR), a key enzyme in chlorophyll biosynthesis (Ma et al., 2014). *MIR171C*-overexpressing plants and *scl6 scl22 scl27* mutants show higher chlorophyll and POR levels than wild-type plants, whereas plants expressing a miR171-resistant form of SCL27 (rSCL27) show reduced chlorophyll and POR levels (Wang et al., 2010; Ma et al., 2014). On the other hand, downregulation of *POR* expression reduces the chlorophyll content of wild-type, *MIR171C*-OX, and *scl* triple mutants (Ma et al., 2014), indicating that the role of miR171c and SCLs on chlorophyll regulation is mediated by PORC. SCL27 represses *PORC* expression by directly binding and inhibiting its promoter.

Moreover, the miR171-*SCL* module also mediates gibberellin-dependent effects on chlorophyll biosynthesis due to its regulation of DELLA proteins and *PORC* expression in the light, but not in the dark. In fact, SCL27 interacts with the DELLA protein RGA and this interaction reduces the ability of SCL27 to bind to the *PORC* promoter. Finally, SCLs induce the expression of *MIR171* genes, revealing the existence of a regulatory feedback loop (Ma et al., 2014). Ma et al. (2014) propose that this feedback loop may also contribute to maintain the diurnal oscillation of miR171.

Taken together, these findings emphasize the role of miRNAs in diverse light-regulated processes. In addition, miRNAs can also directly target major regulators of the light signaling pathways and/or the photoperiod pathway, thus modulating photomorphogenesis or the time to flower, respectively. The most recent findings on miRNAs involved in these processes are discussed below.

#### Photomorphogenesis

Within the group of light-responsive miRNAs, the miR156/157 family occupies a prominent position (**Table 1**). In *Arabidopsis*, miR157d and miR319 promote the degradation of their target transcripts, encoding the positive and negative key photomorphogenesis regulators HY5 and TCP (TEOSINTE BRANCHED1, CYCLOIDEA, and PCF) transcription factors, respectively. Interestingly, both induction and stabilization of miR157d and miR319 were shown to depend on HEN1, with HEN1 accumulation increasing miR157d and miR319 levels in de-etiolating seedlings (Tsai et al., 2014). In turn, HY5 induces *HEN1* expression in a light-dependent manner. Therefore, HEN1 and HY5 constitute a negative feedback regulatory loop that is mediated by miR157, since this miRNA will ultimately target *HY5* transcripts for cleavage. In a *hen1* mutant, both *HY5* transcript and protein levels are increased, probably due to the decrease in miR157d, resulting in a light-hypersensitive phenotype. Oppositely, miR157 constitutive expression reduced *HY5* transcript and protein levels, resulting in seedlings displaying a light hyposensitivity phenotype. Nevertheless, the light-hypersensitive phenotype displayed by *hen1* mutants may also be dependent on miR319, which promoted the cleavage of *TCP* mRNAs and repressed hypocotyl elongation (Tsai et al., 2014). The role of miR319 in photomorphogenesis has been confirmed using a loss of function mutant, which shows longer hypocotyls than the wild type under red light (Sun et al., 2018). Perhaps, the HEN1/HY5 feedback loop may help explain the inconsistencies found in the effect of HEN1, miR156, and miR858 on anthocyanin accumulation.

Three additional miRNAs, miR160, miR167, and miR848, affect *Arabidopsis* hypocotyl elongation under red light (Sun et al., 2018). It is worth mentioning that miR160 and miR167 respond to different light conditions in several species (**Table 1**). Further understanding of the role of these miRNAs in light responses will undoubtedly provide novel insights into how miRNAs contribute to adaptation to different light environments.

Besides the early stages of photomorphogenesis, when the seedling reaches for light and hypocotyl elongation is critical, miRNAs regulate other light responses during plant adult life, such as the perception of their surroundings and neighbors or the time to flower, for instance. When in isolation, plants receive light with a high red to far-red (R:FR) ratio. However, when they grow under a canopy or in close proximity to neighboring plants, the R:FR ratio is lower and this triggers diverse morphological and physiological responses that allow the plants to adapt to these light conditions (Ballaré and Pierik, 2017). When *Arabidopsis* plants grown under light/dark cycles are treated with a pulse of far-red light at the end of the light period (end-of-day far-red, EOD-FR), they show earlier flowering, increased petiole elongation, and a reduction in the number of rosette branches in comparison with plants grown under normal white-light/dark cycles (WL; Xie et al., 2017). EOD-FR treatment causes a decrease in mature miR156 levels by downregulating the expression of several *MIR156* genes. Consistent with this, there is upregulation of several miR156-target *SPL* genes in EOD-FR-treated plants (Xie et al., 2017). This suggests that the miR156-SPL module might be involved in EOD-FR responsiveness. Indeed, when plants with reduced miR156 activity (*MIM156* plants) are grown under WL, they show a constitutive EOD-FR response (Xie et al., 2017). These findings suggest that a reduction in miR156 levels is required for this response, and place miR156 as a repressor of this process.

PHYTOCHROME-INTERACTING FACTORS (PIFs) mediate the effect of phytochromes on numerous light responses (Leivar and Monte, 2014). PIF protein abundance increases under low R:FR light conditions (Lorrain et al., 2008; Leivar et al., 2012; Xie et al., 2017), and PIFs act as positive regulators of EOD-FR responses, showing therefore an opposite behavior to miR156. Further substantiating a previous report suggesting that *MIR156E* is a direct target gene of PIF5 (Hornitschek et al., 2012), Xie et al. (2017) showed that several PIFs repress transcription of five *MIR156* genes, including *MIR156E*, by directly binding to PIF-binding sites present in their promoters. This causes a reduction in mature miR156 levels and a concomitant increase in miR156-target *SPL* transcript abundance. Moreover, genetic analyses show that miR156 acts downstream of PIF5 (Xie et al., 2017). Altogether, these results demonstrate that miR156 responds to EOD-FR treatments and mediates, probably through downregulation of *SPL* genes, the effect of PIFs on at least part of the low R:FR light responses. It will be interesting to determine whether these findings can be reproduced under low R:FR conditions more closely resembling canopy or proximity shade. Therefore, not only do miR156/157 respond to light but also affect several light-regulated processes.

#### miRNAs and Photoperiod-Regulated Processes

Photoperiod affects many plant metabolic, physiological, and developmental processes. It also affects the levels of some miRNAs, as we have already discussed (Li et al., 2015). An example is miR156, which is regulated by photoperiod in potato and soybean, although not in *Arabidopsis* (Jung et al., 2012; Bhogale et al., 2014; Li et al., 2015). miR156, *via* its target genes, downregulates miR172 in several plant species (Chuck et al., 2007; Wu et al., 2009; Bhogale et al., 2014). In *Arabidopsis*, mature miR172 levels are higher under LD than SD and this difference does not seem to be driven by transcription, given that the abundance of at least two miR172 primary transcripts is lower under LD than SD (Jung et al., 2007). Rather, it probably results from regulation of miR172 processing. A *gigantea* (*gi*) mutant, which shows a reduced response to photoperiod and reduced expression of two genes involved in miRNA processing, *DCL1* and *SE*, shows lower mature miR172, but not *pri-miR172*, levels than the wild type. However, additional factors must contribute to the photoperiodic regulation of miR172, given that this miRNA still responds to photoperiod in the *gi* mutant (Jung et al., 2007). Potato and soybean also show different miR172 levels under LD and SD (Martin et al., 2009; Li et al., 2015), suggesting that the photoperiodic regulation of this miRNA may be evolutionarily conserved.

Interestingly, at least four photoreceptors, phytochrome A, phytochrome B, and cryptochrome 1 and 2, regulate miR172 levels (Jung et al., 2007; Martin et al., 2009; Zhao et al., 2015). In agreement with this, red light downregulates miR172 and blue light upregulates it in *Arabidopsis*. Therefore, both light quality and duration affect miR172 abundance. In addition, TIMING OF CAB EXPRESSION1 (TOC1), a component of the circadian clock, reduces mature miR172 levels, but despite this, mature miR172 levels do not show daily oscillations in *Arabidopsis* (Jung et al., 2007). Further research is still needed to determine whether the circadian clock regulates miR172.

**FIGURE 3:**
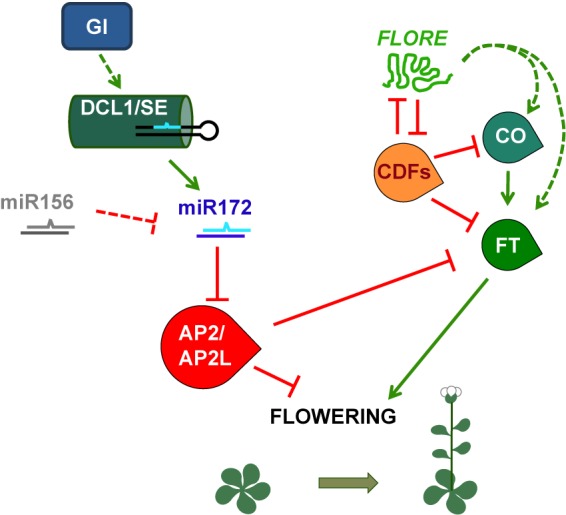
Simplified model of the photoperiodic regulation of *Arabidopsis* flowering time mediated by the miRNA miR172 and the lncRNA *FLORE*. GI regulates the DCL1/SE complex and thus miR172 accumulation, which will in turn modulate the protein levels of the flowering repressors AP2 and AP2-like. On the other hand, light-responsive miR156 is able to repress miR172. Although miR156 is regulated by photoperiod in soybean and potato, in *Arabidopsis* this regulation in not clear. AP2 and AP2-like exert their function by inhibiting *FT* transcription, which is positively regulated by CO. The CO-FT module is also negatively targeted by the CDFs. In addition, *FLORE* is able to inhibit these transcription factors and thus promote an increase in *CO* and *FT* transcript levels, most likely in an indirect way. This model provides a glimpse of the different layers of regulation required to ensure that *Arabidopsis* plants flower under the most favorable photoperiodic conditions, which occur when day length increases and short days give way to longer days.

In *Arabidopsis*, flowering is regulated by photoperiod, such that plants flower earlier under LD than SD. By contrast, soybean flowering and potato tuber formation are induced by SD. Overexpression of miR172 promotes flowering in *Arabidopsis* and tuberization in potato and reduces the photoperiodic tuberization response (Aukerman and Sakai, 2003; Chen, 2004; Jung et al., 2007; Martin et al., 2009), strongly suggesting that miR172 is involved in the regulation of photoperiodic processes. miR172 targets a subfamily of *AP2*-like genes, including *AP2* itself, which partially redundantly repress flowering (Zhu and Helliwell, 2011). In *Arabidopsis*, and probably in soybean, alteration of miR172-target gene expression or function leads to a reduced response of flowering to photoperiod, confirming the role of the miR172/AP2-like module in the photoperiodic regulation of flowering (Mathieu et al., 2009; Yant et al., 2010; Zhao et al., 2015). In *Arabidopsis* plants grown under LD, GI, and probably several photoreceptors, upregulates miR172, which negatively regulates its target genes, which in turn directly repress the expression of several genes promoting flowering, including *FLOWERING LOCUS T* (*FT*), and promote the expression of genes repressing flowering (**Figure 3**) (Jung et al., 2007; Mathieu et al., 2009; Yant et al., 2010). Downregulation of miR172 target genes, thus, accelerates flowering under LD conditions. This photoperiodic flowering pathway is independent of the photoperiodic flowering regulator CONSTANS (CO) in *Arabidopsis* (Jung et al., 2007; Mathieu et al., 2009; Yant et al., 2010), although it has been proposed to involve a *CO-like* gene in soybean (Zhao et al., 2015).

Given that miR156 responds to light in several species, to photoperiod in potato and soybean, is involved in flowering control, and negatively regulates miR172 (Chuck et al., 2007; Wang et al., 2009; Wu et al., 2009; Bhogale et al., 2014; Li et al., 2015) (**Table 1**), it seems likely that miR156 may play a role in photoperiodic flowering. However, whether this is the case still needs to be proved. In addition to light quality and photoperiod, miR156 and miR172 levels respond to other environmental and endogenous signals and play roles in processes other than flowering and tuberization (Zhu and Helliwell, 2011; Han et al., 2013; Nova-Franco et al., 2015; Ripoll et al., 2015; Wang and Wang, 2015; Huo et al., 2016; Li et al., 2017; Díaz-Manzano et al., 2018; Luan et al., 2018), indicating that miR156 and miR172 influence many aspects of plant biology.

The miR170/171 family has been shown to be responsive to different light conditions in several species (**Table 1**). In addition, the rice *OsMIR171C* promoter contains several elements putatively involved in light responses (Fan et al., 2015). Consistent with this, *OsMIR171C* transcript levels show a diurnal oscillation with a peak early in the morning, similar to the oscillation of mature miR171 in *Arabidopsis* (Fan et al., 2015). Conversely, transcript levels of four rice miR171 target genes, *OsHAM1* to *OsHAM4*, accumulate from the evening until early morning under light/dark cycles. Although it is not clear whether these rice oscillation patterns are regulated by light or by the circadian clock, miR171c levels respond to photoperiod, with higher levels under LD than SD (Fan et al., 2015). miR171c upregulation in the *delayed heading* (*dh*) mutant, which carries a T-DNA insertion in the *OsMIR171C* promoter, leads to late flowering (heading), strongly suggesting that miR171c represses flowering (Fan et al., 2015). Moreover, the upregulation of miR171c by LD, which are non-inductive conditions for rice flowering, fits with a role in delaying flowering. However, further research is still required to determine if miR171 affects the photoperiodic regulation of flowering in rice. Nevertheless, the expression pattern of miR171c and *OsHAMs* in the shoot apex of wild-type plants is consistent with a role in regulating the floral transition. This is also supported by the downregulation of three key positive regulators of flowering (*Ehd1, Hd3a*, and *RFT1*) in the *dh* mutant in comparison with the wild type (Fan et al., 2015). Interestingly, the *dh* mutant also shows upregulation of miR156 (Fan et al., 2015), a flowering repressor miRNA in several species (Wang, 2014), and of *OsPHYC*, which encodes a photoreceptor that also delays flowering in rice (Takano et al., 2005; Fan et al., 2015). Therefore, miR171 is regulated by light and, in turn, it may be involved in regulating light responses mediated by OsPHYC.

Another miRNA that responds to day length and regulates photoperiodic flowering is miR5200, a miRNA specific of the *Pooideae* family. In a *Brachypodium distachyon* accession that flowers much earlier under LD than SD, mature miR5200, as well as *pri-miR5200a* and *pri-miR5200b*, is present at much higher levels under SD than LD and its pri-miRNAs follow a daily rhythm under SD (Wu et al., 2013). The expression pattern of *MIR5200A* and *MIR5200b* correlates with active histone marks in these genes under SD and repressive histone marks under LD. Another five *Pooideae* species accumulate more miR5200 under SD than LD, revealing conservation of this photoperiodic regulation among these grass species (Wu et al., 2013). In *B. distachyon*, miR5200 targets two *FT*-like genes, *FTL1* and *FTL2*, whose transcripts are more abundant under long than short photoperiods. *B. distachyon* plants overexpressing this miRNA flower much later than wild-type plants under LD, whereas plants with reduced miR5200 activity (*MIM5200*) flower earlier than the wild type under SD, and like the wild type under LD, showing therefore a reduced response to photoperiod. Consistently, *FTL1* and *FTL2* mRNA levels are increased in *MIM5200* plants under SD, but unchanged under LD in comparison with wild-type plants (Wu et al., 2013). Therefore, miR5200 plays an important role in the photoperiodic regulation of flowering in *B. distachyon* by delaying flowering under SD.

## Long Non-Coding RNAs

Next-generation sequencing techniques have unraveled a whole new transcriptional landscape that includes lncRNAs. Several screening approaches have been designed to identify novel plant lncRNAs that would accumulate in response to certain environmental cues, in specific organs/tissues or at particular developmental stages. However, although lncRNA listings are available, the functional characterization of most of these transcripts is yet to be done. Most of the knowledge on plant lncRNA function is associated with *FLOWERING LOCUS C* (*FLC*), a negative regulator of flowering. In fact, several lncRNAs arising from this *locus* were shown to repress *FLC* transcript levels upon vernalization and thus promote flowering. *COLD OF WINTER-INDUCED NON-CODING RNA FROM THE PROMOTER* (*COLDWRAP*) is transcribed from the *FLC* promoter region, whereas *COLD-ASSISTED INTRONIC NON-CODING RNA* (*COLDAIR*) is expressed from the first intron. *ANTISENSE LONG* (*ASL*) and *COLD-INDUCED LONG ANTISENSE INTRAGENIC RNA* (*COOLAIR*) both originate from the *FLC* 3′*UTR* region and show partial overlap. The combined action of the three lncRNAs was shown to silence *FLC* expression, and therefore to promote flowering, through the accumulation of repressive histone marks (reviewed in Chekanova, 2015; Whittaker and Dean, 2017). Although most of the research on these lncRNAs pertains to vernalization-regulated flowering, and therefore is outside the scope of this review, there are reports connecting *FLC* with photoperiod and circadian regulation. *FLC* lengthens the circadian period, especially at high temperature (Swarup et al., 1999; Edwards et al., 2006), but shortening of the circadian period by vernalization is not mediated by *FLC* (Salathia et al., 2006). Therefore, whether the regulation of *FLC* by vernalization-associated lncRNAs is linked to its role in circadian periodicity is yet to be determined.

Several *FLC* clade members were shown to regulate flowering both under LDs and SDs. Protein levels of one of them, MAF3 oscillated throughout the day, suggesting regulation by the photoperiod or the circadian clock (Gu et al., 2013). In fact, FLC clade members could form multi-protein complexes and regulate flowering depending on photoperiod and temperature. However, further research is needed to determine whether vernalization-associated lncRNAs would also participate in these processes. Therefore, in this section, we will discuss the available reports focused on light and circadian-responsive lncRNAs.

### lncRNAs as Regulators of Light-Dependent Processes

One of the screening approaches that have allowed the identification of novel lncRNA transcriptional units used a reproducibility-based tiling-array analysis strategy (RepTAS) previously described by Liu et al. (2012). Thus, Wang H. et al. (2014) identified a total of 37,238 light-responsive lncNAT pairs (NAT pairs where one of the components is an lncRNA) in *Arabidopsis*. These NAT pairs were afterward verified by different methods, including the screening of a novel ATH (*Arabidopsis thaliana*) NAT custom array, strand-specific RNA-seq (ssRNA-seq), and quantitative RT-PCR (RT-qPCR). To identify *bona fide* light-regulated lncRNAs, the expression levels of these antisense and sense transcripts were determined in etiolated seedlings and seedlings undergoing de-etiolation that were exposed to continuous white light for 1 and 6 h. Not only did this approach allow the identification of light-responsive lncNATs but also revealed their preferential accumulation in cotyledons compared to hypocotyls, and roots. Interestingly, the number of light-responsive lncNAT pairs was much higher after 6 h of treatment than at 1 h, indicating spatial-, developmental-, and temporal-specific patterns of accumulation. When the expression levels of both the sense and antisense components of each NAT pair was determined, these lncNAT pairs were classified into two groups: light-responsive concordant NAT pairs and light-responsive discordant NAT pairs. In order to further characterize their expression pattern, a parallel analysis was performed matching specific histone modifications with their transcriptional changes. This approach showed that transcription changes of these light-regulated NATs were preferentially correlated with histone H3 acetylation (H3K9ac and/or H3K27ac), H3K9ac being the predominant positive histone mark, suggesting its involvement in regulating light-responsive NAT pairs (Wang H. et al., 2014). Although the functional characterization of most of these candidates is yet to be done, these results provide the first evidence that light is a major shaper of the plant non-coding transcriptional landscape, including both long and small non-coding RNAs.

*HIDDEN TREASURE 1* was the first lncRNA described as a positive regulator of photomorphogenesis under continuous red light. This is achieved by *HID1* ability to downregulate the expression levels of *PIF3*, a negative regulator of this process (Wang Y. et al., 2014). In agreement with this, *PIF3* mRNA levels were increased in *hid1* mutants that displayed elongated hypocotyls under red light, a typical phenotype of PIF accumulation. Functional characterization of *HID1* revealed that this lncRNA assembles in nuclear protein–RNA complexes where it is able to associate with *PIF3 5*′*UTR* and repress its expression. Therefore, *HID1* would act through *PIF3* to modulate hypocotyl elongation. Sequence homology searches revealed that *HID1* is present in other plant species. Confirming its conservation, *Arabidopsis hid1* mutants expressing *OsHID1*, a rice homolog, could be rescued of their elongated hypocotyl phenotype (Wang Y. et al., 2014). These results suggest that lncRNAs may be conserved between species and regulate similar biological processes, similar to the families of conserved miRNAs.

Interestingly, besides its role as a positive regulator of photomorphogenesis in red light, *HID1* can also act as a negative regulator of cotyledon greening during de-etiolation of *Arabidopsis* in white light (Wang et al., 2018). Within the several steps of this process, the reduction of protochlorophyllide (Pchlide) to chlorophyllide *via* POR is fundamental for cotyledon greening to occur. Confirming its repressor role in this process, dark-grown *hid1* mutants transferred to white light presented a higher rate of cotyledon greening compared to *Arabidopsis* wild-type plants. Moreover, *HID1* knockdown led to a reduction of Pchlide content in the dark, as well as higher *POR* mRNA levels, indicating that *HID1* plays a role in cotyledon greening by promoting Pchlide accumulation and repressing *POR* transcription (Wang et al., 2018). To uncover the *HID1*/*PIF3* relationship in cotyledon greening, double *hid1 pif3* mutants were generated. *PIF3* was previously shown to regulate seedling greening by preventing Pchlide over-accumulation (Leivar and Monte, 2014). However, *hid1 pif3* double mutants greening rate was comparable to that of *hid1*, suggesting that *HID1* would act downstream of *PIF3* (Wang et al., 2018). Since PIF3 does not regulate *HID1* expression, the exact mechanism by which *HID1* and PIF3 regulate greening still remains to be determined (Wang et al., 2018). Together, these results suggest that depending on the light-mediated developmental process, *HID1* could associate with different factors allowing it to perform different functions.

### Circadian-Regulated lncRNAs in Photoperiod-Dependent Flowering

Long non-protein coding RNAs can also display circadian regulation. This is characterized by their ability to maintain an oscillatory pattern of expression even in the absence of environmental cues (e.g., under constant conditions). Several screening approaches allowed the identification of circadian ncRNAs in fungi, mammals, and *Arabidopsis*, some of them being lncRNAs that were also NATs (Kramer et al., 2003; Hazen et al., 2009; Coon et al., 2012; Xue et al., 2014). However, even if non-coding NATs were reported for components of the *Arabidopsis* oscillator (Hazen et al., 2009), their function had not been investigated. Recently, we characterized a circadian-regulated *Arabidopsis* lncRNA, *FLORE*, which is a NAT to the *CYCLING DOF FACTOR 5* (*CDF5*) transcriptional regulator (Henriques et al., 2017). *FLORE* and *CDF5* displayed an antiphasic pattern of expression that resulted in part from a mutual inhibitory function, as well as an opposite biological function (**Figure 3**). CDFs have been characterized as repressors of photoperiodic flowering due to their ability to repress *CO* and *FT* transcription (Song et al., 2015). *FLORE* inhibits *CDF5* accumulation and contributes to maintain its proper oscillatory pattern. Therefore, due to downregulation of *CDF5, FLORE* overexpression in the vascular tissue promotes flowering. On the other hand, *CDF5* will also regulate *FLORE* transcript levels, thus contributing to its oscillation. These findings indicate that the circadian clock is able to control the expression of both coding and non-coding transcripts, in this case, *CDF5* and *FLORE* (Henriques et al., 2017). However, the mutual regulation within this NAT pair seems to be required to ensure the timely accumulation of each transcript. Further research is still necessary to uncover the molecular mechanisms that account for this regulation.

## Final Conclusions

Both small and lncRNAs have been associated with specific biological processes that can occur at particular developmental stages, often in very precise locations within certain organs and tissues. Light is one of the major environmental cues for plants and essential for their survival. Therefore, it is not surprising that light modulates the expression, processing, and activity of these non-coding RNAs. It became apparent that, in the case of specific miRNAs, a correlation pattern emerges where the accumulation of specific families associates with particular light conditions in different species. Moreover, this seems to be a two-way regulation since miRNAs can also target basic components of light and photoperiod signaling, thus being also required for the proper functioning of these pathways. Although some of the miRNAs that are consistently revealed as light-regulated in diverse plant species have not been shown to play functional roles in light responses so far, they are excellent candidates for performing such roles. lncRNAs have recently become the subject of scrutiny, but the few reports available prevent the establishment of any specific trend. Sequencing and bioinformatics efforts led to the construction of a plant-specific lncRNA database (Jin et al., 2013). However, other approaches combining transcriptomics, epigenetic, and functional assays would provide further insights into the biological role of plant lncRNAs. Nevertheless, it is noteworthy mentioning that, from the reduced group of studied lncRNAs, two of them associate with light-dependent processes. These findings do show, however, that light is a massive re-programmer of non-coding transcription able to set in motion-specific fine-tuning mechanisms with the ultimate goal of adjusting plant development to their surroundings.

## Author Contributions

All the authors read the cited publications, prepared summaries, and wrote the manuscript.

## Conflict of Interest Statement

The authors declare that the research was conducted in the absence of any commercial or financial relationships that could be construed as a potential conflict of interest.
